# Effect of 15% carbamide peroxide bleaching gel on color stability of giomer and microfilled composite resin: An in vitro comparison

**DOI:** 10.4317/medoral.17916

**Published:** 2012-08-28

**Authors:** Narmin Mohammadi, Soodabeh Kimyai, Mehdi Abed-Kahnamoii, Mohammad-Esmaeel Ebrahimi-Chaharom, Alireza Sadr, Mehdi Daneshi

**Affiliations:** 1 DDS, MS, Associate Professor, Department of Operative Dentistry, School of Dentistry, Tabriz University of Medical Sciences, Tabriz, Iran; 2 DDS, MS, Assistant Professor, Department of Operative Dentistry, School of Dentistry, Tabriz University of Medical Sciences, Tabriz, Iran; 3 DDS, PhD, Junior Associate Professor, Department of Cariology and Operative Dentistry, Global Center of Excellence, IRCMSTBD, Tokyo Medical and Dental University, Tokyo, Japan; 4 DDS, Post-graduate student, Department of Operative Dentistry, School of Dentistry, Tabriz University of Medical Sciences, Tabriz, Iran

## Abstract

Objectives: The effect of 15% carbamide peroxide bleaching gel on color stability and surface topography of a giomer and a microfilled composite resin was evaluated in the present in vitro study. Study design: Forty discs measuring 10 mm in diameter and 1 mm in thickness were prepared from a giomer and a microfilled composite resin. Each material yielded 20 discs with completely smooth surfaces. Then a spectrophotometer was used to measure L* (lightness), a* (redness, greenness) and b* (blueness, yellowness) color coordinates of all the discs. Subsequently, the specimens were subjected to 15% carbamide peroxide bleaching gel. After measuring the color coordinates once again, color changes (ΔE*) were calculated by the CIELAB system. Six specimens from each material (three specimens before bleaching agent application and three specimens thereafter) were viewed under an atomic force microscope (AFM) for surface topography evaluation. Data were analyzed by Mann-Whitney U and Kruskal-Wallis tests at α=0.05. Results: There were no statistically significant differences in color changes (ΔE*) between the two materials (P>0.05). In addition, no significant differences were detected in surface roughness between composite resin and giomer discs before and after bleaching (P>0.05 for both). However, in both materials the differences in surface roughness were significant before and after bleaching procedures (P<0.001). Conclusions: Based on the results of the present study it was concluded that 15% carbamide peroxide does not induce clinically detectable color changes in composite resin and giomer despite an increase in surface roughness.

** Key words:**Bleaching, color stability, giomer, microfilled composite.

## Introduction

The use of peroxide-containing tooth bleaching agents has increased in the recent decade with the advent of at-home bleaching technique (1). Although bleaching agents improve the esthetic appearance of bleached teeth, their contact with tooth-colored restorations might induce changes in physical and chemical properties of the restoration due to its softening effect (2,3). In addition, bleaching agents can induce discolorations in tooth-colored dental materials (4,5). Discoloration of tooth-colored restorations in the oral cavity is considered to be one of the most important disadvantages of such restorations, and is the main reason for their replacement (3).

Typically, microfilled composite resins are the materials of choice for Class V restorations (6,7). Some studies have evaluated the color stability of composite resins after they contact bleaching agents. Hubbezoglu et al (8) reported that 16% and 30% carbamide peroxide bleaching gels had no effect on the color of microfilled composite resins while 35% hydrogen peroxide resulted in significant discoloration. On the other hand, the results of a study by Rao et al (9) showed that among three restorative materials, glass-ionomer cement, microfilled composite resin and nanofill composite resin, bleached by three different concentrations of carbamide peroxide gel, glass-ionomer cement and microfilled composite resin show the most severe discolorations.

Recently a new type of composite resin has been introduced, which is referred to as giomer or pre-reacted glass-ionomer (PRG) composite. A two-year clinical trial has shown that this material might successfully be used for Class V restorations (10). Giomer consists of PRG particles which are placed in a resin matrix as fillers (10). These materials have fluoride release and recharge capacities (11). Since no studies to date have evaluated the effect of bleaching agents on color stability of giomers, the present in vitro study was undertaken to evaluate the effect of 15% carbamide peroxide bleaching gel on color stability of a giomer and a microflled composite resin. The null hypothesis was that 15% carbamide peroxide bleaching gel has no effect on color stability or roughness of giomer and resin composite.

## Material and Methods

In the present in vitro study, a microfilled composite resin (Heliomolar; Ivoclar Vivadent, Schaan, Liechtenstein) and a giomer (Beautifil II; Shofu Dental Corporation, Osaka, Japan) were used. Chemical compositions of the two materials are presented in  Table 1. Twenty specimens were prepared using A3 shade of each material. In order to prepare the specimens, plastic molds, with an inner diameter of 10 mm and a height of 1 mm, were used. The molds were placed on glass slabs and the materials were packed into the molds. Transparent matrix bands were placed on the materials to achieve a smooth homogenous surface; then a glass slab was pressed on each mold containing the material. The specimens were light-cured through the 1-mm glass slide using a light-curing unit (Astralis 7; Ivoclar Vivadent, FL-9494 Schaan, Liechtenstein) at a light intensity of 700 mW/cm2 for 40 seconds. The tip of the light-curing unit was in contact with the glass slide during polymerization. The specimens were removed from the molds and were again light-cured from four directions for 20 seconds each to ensure proper polymerization (7). Subsequent to storage of the specimens in distilled water for 24 hours at 37ºC for complete polymerization, the upper surfaces of the specimens were polished with fine and super-fine Sof-Lex discs (3M ESPE Dental Products, St. Paul, MN, USA) to achieve uniform and smooth surfaces and remove all possible contaminants and the oxygen-inhibited layer (3). Prior to the initial measurement of color coordinates, the specimens were rinsed under tap water for 1 minute and bottled dry (8).

**Table 1:**
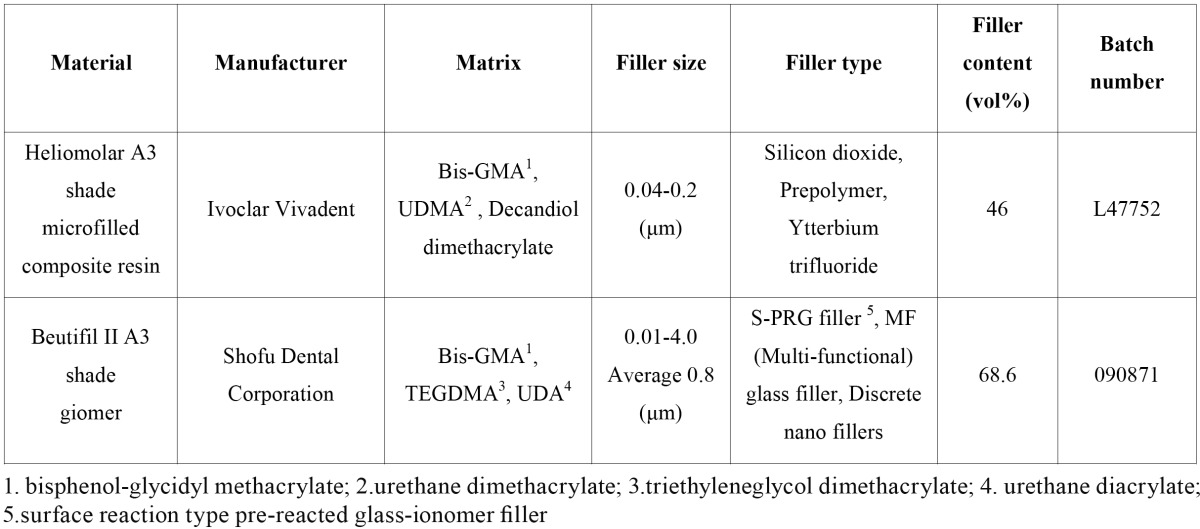
Chemical compositions of composite resin and giomer used in the study.

The color coordinates were determined with the use of a reflexive spectrophotometer (Color-Eye 7000A; CretagMacbeth, New Windsor, NY, USA) under D65 light source in a white background. This device has the capacity to measure color in a manner in which it matches the geometry of visual assessments; it has been used in some previous dental research studies (3,4). Before measurement sessions, the spectrophotometer was calibrated using the standards supplied by the manufacturer. The geometric condition (illumination/measurement) for colorimetry measurements was 45º/d, and 10° CIE standard observer was used to calculate color.

The specimens were separately placed in numbered containers in distilled water. The color coordinates measured were L* (lightness), a* (redness, greenness) and b* (blueness, yellowness). Each specimen was measured twice by the same operator and the average values of L*, a* and b* were calculated.

Subsequently, the specimens were immersed in 15% carbamide peroxide bleaching gel (Ultradent Products, South Jordan, UT, USA) for eight hours daily for 14 days to simulate at-home bleaching technique (1,3). Subsequent to a daily bleaching procedure, the specimens were rinsed under tap water for 1 minute to remove the bleaching agent, bottled dry and stored in distilled water at 37ºC. The bleaching agent was replenished every day. After the 14-day period, the color coordinates were measured again and the color differences were calculated by the following formula according to Commision Internationale de l’Eclairage System (1,3):

ΔE*=[(ΔL*)2+(Δa*)2+(Δb*)2]1/2.

Three additional specimens from each of the four experimental groups were prepared for the evaluation of surface topography under an atomic force microscope (AFM) (NanoScope II; Digital Instruments, Santa Barbara, CA, USA). To this end, four quadrants from each specimen were evaluated. For the AFM procedure a silica nitride tip (with a nominal radius of 50 nm, apex angle of 45°, height of 2.8 μm, and a base of 4×4 μm) was used. The images were recorded with a scan rate of 1.9 Hz and a resolution of 256×256 pixels per image over an area of 5 µm × 5 µm. Four images were produced from each specimen prepared for AFM evaluation; therefore, 12 images were evaluated under AFM on the whole. Three-dimensional topographic images were produced by AFM and analyzed by NanoScope III software (Version 5.12r2, Digital Instruments, Santa Barbara, CA, USA). Surface roughness of each specimen was calculated in nm as the root mean square (rms) value of surface departures in each sampling area. Surface roughness (nm) and surface area differences in relation to the smoothest condition (%) were determined for all the specimens.

Data were analyzed by non-parametric Mann-Whitney U and Kruskal-Wallis tests using SPSS /Win.15. Statistical significance was defined at α=0.05.

## Results

-Color stability

Descriptive statistics [means ± standard deviations (SD)] in relation to color coordinates and color stability (ΔE*) in both groups are presented in  Table 2.

**Table 2:**
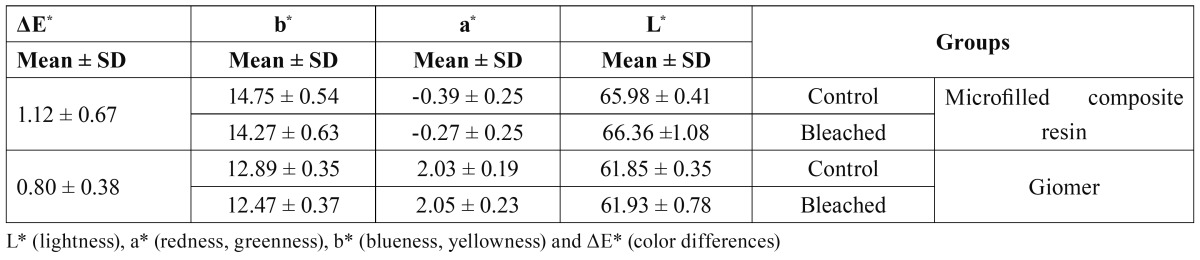
Color stability descriptive statistics.

The results of the Mann-Whitney U test did not demonstrate any significant differences in color stability between the two groups (U=142, P=0.12).

There were statistically significant differences in L* and b* values subsequent to bleaching [(U=123, P=0.03) and (U=122, P=0.03), respectively] in microfilled composite resin; however, the differences in a* values subsequent to bleaching were not statistically significant (U=135, P=0.08).

There were statistically significant differences in b* values subsequent to bleaching (U=88.50, P=0.02) in giomer restorative material; however, the differences in L* and a* values subsequent to bleaching were not statistically significant [(U=170.50, P=0.42) and (U=200, P=1.00), respectively].

-Surface roughness

 Table 3 presents descriptive statistics and the results of comparative tests between the two groups in relation to surface roughness.

**Table 3:**
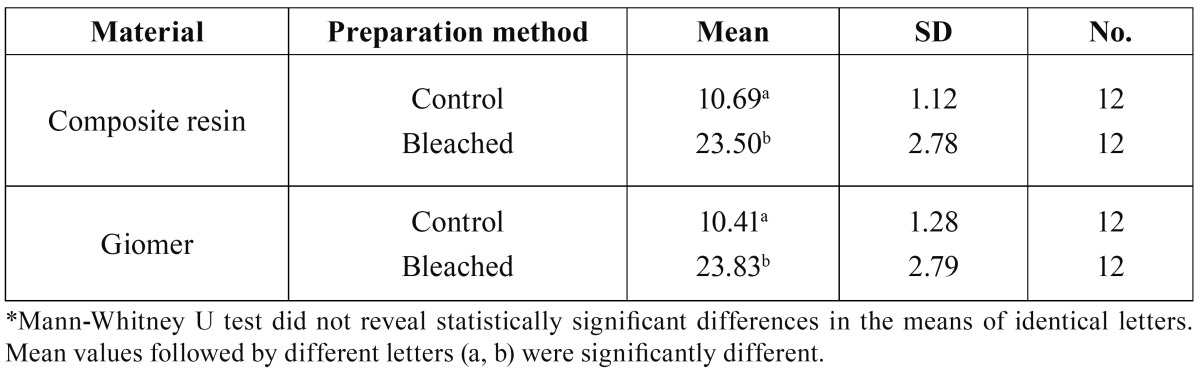
Means and standard deviations (SD) of surface roughness [Rms, nm] values in the groups under study*.

Kruskal-Wallis test revealed statistically significant differences in surface roughness between the groups under study (X2=35.66, df=3, P<0.001).

Two-by-two comparison of the groups using Mann-Whitney U test revealed statistically significant differences in surface roughness values in composite resin before and after bleaching (P<0.001), and also in giomer before and after bleaching (P<0.001). However, there were no significant differences in surface roughness values between the two materials before and after bleaching (P=0.59 and P=0.40, respectively).

-Surface area differences under AFM

 Table 4 represents descriptive statistics of surface area differences and the results of comparisons between the groups under study.

**Table 4:**
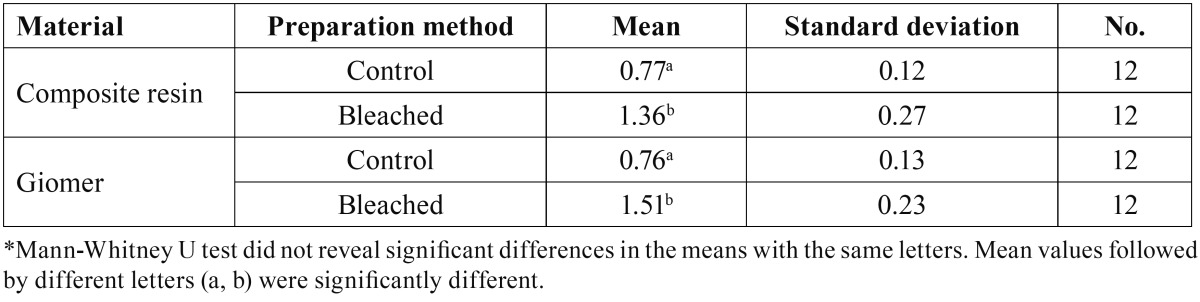
Means and standard deviations in relation to regional surface changes [%] in the groups under the study*.

Kruskal-Wallis test revealed statistically significant differences in the means of surface area differences (X2=36.06, df=3, P<0.001). Two-by-two comparison of the groups by Mann-Whitney U test revealed significant differences between surface area differences in giomer before and after bleaching (P<0.001) and also in composite resin before and after bleaching (P<0.001). However, there were no significant differences in the surface area differences between the two materials before and after bleaching (P=0.77 and P=0.12, respectively).

**Figure 1:**
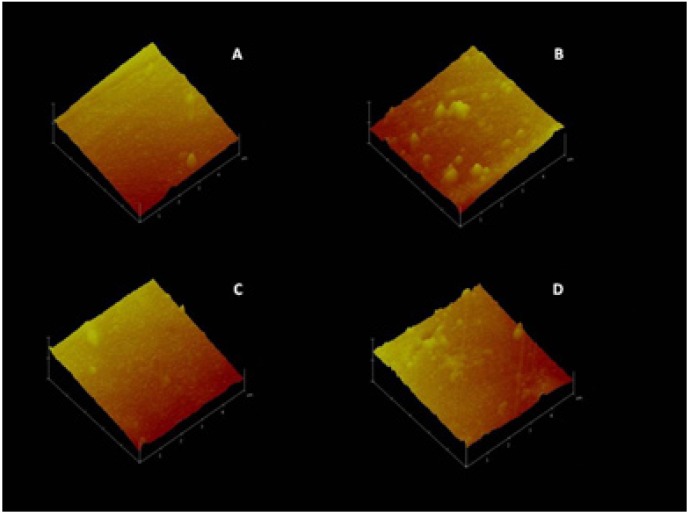
Three-dimensional topographic images of composite resin and giomer surface produced by AFM: A) (composite resin, control); B) (composite resin, bleached); C) (giomer, control); D) (giomer, bleached).

Figure 1 shows surface topography in the study groups under AFM.

## Discussion

The effect of tooth bleaching agents on tooth-colored restorative materials is of clinical interest and several studies have evaluated the effect of commonly used bleaching agents on a number of restorative materials. Since evaluation of color changes by photometric equipment is much more accurate than that by the naked eye, the CIELAB technique was used to this end in the present study. In this technique, the color of specimens is measured by spectrometry in a white background (1,8,9). Although some recent studies have reported that CIEDE2000 color difference formula (ΔE00) furnishes a better fit compared to the CIELAB formula (ΔE*ab) in evaluation of color differences in the dental field, the majority of newly published papers still make references to CIELAB formula (12-14), which was attributed by Ghinea (13) to the relative complexity of the ΔE00 formula and the ease and simplicity of comparison with previous studies. As a result, ΔE*ab formula was used in the present study.

In previous studies the thickness of the composite specimens was 2 mm. It has been reported that the color chan-ges presumably become more noticeable if the thickness of the specimens decrease (1,2,8). Therefore, the specimens with a thickness of 1 mm were used in this study.

The results of the present study did not reveal any significant differences in color stability of Heliomolar microfilled composite resin and Beautifil II giomer under the influence of 15% carbamide peroxide bleaching gel. Generally, two key factors determine the extent of the effect of peroxide-containing materials: peroxide concentration and application duration (8). In this study, both factors were the same for both materials: 15% carbamide peroxide was used 8 hours daily for two weeks.

Several studies have reported the perceptibility and/or acceptability of dental color differences. Some investigations have reported that color changes of more than 1 (ΔE*>1) are discernible by approximately 50% of people by the naked eye. In addition, ΔE*≥3.3 is clinically unacceptable and the restoration should be replaced (15,16). In all-ceramic crowns, a color difference of ΔE*=1.60 could not be detected by human eye (17). In a recent study, Ghinea et al (12) established ΔE*=3.48 and ΔE*=1.80 units as new 50:50% acceptability and perceptibility thresholds for dentistry, respectively.

In the present study, means of ΔE* for Beautifil giomer and Heliomolar microfilled composite resin were 0.80 and 1.12. Considering the recent thresholds (12) color differences of microfilled composite and giomer subsequent to bleaching were within the acceptability threshold. Moreover, the color difference values for both restorative materials were below the perceptibility threshold. The results of the present study regarding the microfilled composite resin color stability are consistent with those of a study by Hubbezoglu et al (8).

During disintegration process, most of the carbamide peroxide is converted into hydrogen peroxide and the rest into urea. Urea further breaks down into ammonia and carbon dioxide (1,8). Hydrogen peroxide is a strong oxidative agent, which, in turn, is converted to water, oxygen and free radicals. These free radicals whiten the teeth by oxidating the pigments responsible for tooth discoloration (18). Although there were no significant differences in color stability between the two materials, giomer showed slightly less color changes subsequent to bleaching when compared to the mircofilled composite, with no statistically significant difference, which might be attributed to differences in the general structures of the two materials since the type and concentration of the bleaching agent were similar and the same procedure was used for specimens in the two groups under study. It has been reported that the volume of resin matrix and filler type have a greater influence on the color parameters of composites than the structure of the organic matrix (8).

Both materials have a composite structure, i.e. they are composed of three main components of resin matrix, fillers and the coupling agent. Based on the data provided by the manufacturer ( Table 1), the fillers of the microfilled composite are composed of silicon dioxide and Ytterbium trifluoride. This kind of microfilled composite resin is “inhomogeneous”, i.e. it has two types of fillers, which consist of 0.04–0.2 µm silicone particles and organic filler particles, which are bigger so that a maximum filling of the resin matrix would be achieved, resulting in improved mechanical properties. In this regard, the resin content of the composite resin decreases. The filler content of this kind of composite resin is 46 vol% and 66.7 wt% (19). The resin component of Beautifil II giomer is composed of Bis-GMA, UDA and TEGDMA (20). On the other hand, Beautifil II is the second generation of light-cured giomers, with a capacity to release fluoride. Its fillers are composed of glass particles, S-PRG fillers (pre-reacted glass fillers with surface reaction) and discrete nano fillers. S-PRG fillers are in fact glass-ionomer powder particles, which have been activated by polyacrylic acid on the surface (10). A satisfactory esthetic appearance and color matching has been reported using this class of materials even after eight years of clinical service (20). Moreover, Beautifil II giomer contains discrete nano fillers (10-20 nm), in addition to larger particles of up to 4 µm size, which make it possible to incorporate larger filler content of 68.6 vol% and 83.3 wt%. In other words, it has a lower resin content compared to Heliomolar composite resin. As it was previously pointed out, hydrogen peroxide is a strong oxidative agent, which can destroy the resin matrix of composite resin and induce discolorations by influencing their amine and unsaturated components (8). Since the microfilled composite resin has a higher resin content compared to giomer, it may be more susceptible to discoloration in the longer term. In addition to resin and filler content, it has been reported that other filler attributes such as optical properties, shape and size influence the optical properties of restorative materials (21-23). Nevertheless, it has been suggested that different filler particles used in dental composites have close refractive indices; therefore, the influence of other physical factors, including shape, size and content would be dominant on the color of composite (24). In addition, it has been suggested that bleaching agents may also affect the filler particles through dissolution and leaching of ions such as silicon, barium and strontium. Nevertheless, these effects may be negligible when compared to water (25).

Another factor involved in discoloration is degree of conversion of resin matrix (18). Both materials in this study are composed of bisphenol-glycidyl methacrylate (Bis-GMA). It has been reported that in Bis-GMA-based composites triethyleneglycol dimethacrylate (TEGDMA) molecule, similar to that in giomer, behaves as a cross-linking agent and leads to an increased double bond conversion, when compared to a mixture of Bis-GMA and urethane dimethacrylate (UDMA), as in the microfilled composite (26). However, further studies are required to confirm a possible difference in the degree of conversion between the two materials.

In addition, the results of the present study revealed a significant increase in surface roughness and surface area in each material after bleaching, but the differences between the two materials were not significant. In previous studies increased surface roughness and cracks, scratches and small pores under scanning electron microscope after application of carbamide peroxide bleaching gel have been reported in composite resins (1,2,8,18). It has been shown that the free radicals released from bleaching agents can penetrate into the filler-resin bond interface and disrupt the bond (8,18). As a result, microscopic cracks are formed on the surface, leading to an increase in surface roughness and, as a result, in surface area. It has been suggested that an increased surface roughness can lead to an increased adherence of some cariogenic microorganisms to the surface of the restorative materials subsequent to bleaching (18). Therefore, polishing of these restorative materials after bleaching is advisable. Nevertheless, a recent study suggested that bacterial adherence in giomer was lower compared to composite resin groups despite their similar roughness values, which was attributed to the composition of giomer (7).

In previous studies the effect of surface roughness on color of the dental-resin composites has been shown (13,27). The degree of scattering or reflection of the light rays incident on the restorative material is under the influence of surface texture (27). In this study, despite the significant increase in surface roughness of both restorative materials subsequent to bleaching, the color differences were within the acceptability thresholds mentioned above. The differences in the results of different studies might be attributed to different methodologies and materials involved; in two previous studies, the effect of surface roughness induced by silicon carbide papers (with different grits) on color differences of resin composites was evaluated (13,27). Meanwhile, it should be noted that current findings revealed that color coordinates changed as the surface roughness changed subsequent to bleaching. L* is a measure of illuminated reflectance and bleaching resulted in a significant increase in L* values of microfilled composite resin; however, in giomer L* values were not statistically different followed by bleaching despite the significant increase in surface roughness induced by bleaching in both materials. These results are in line with a previous study (28), demonstrating increased reflectance from the surface of a microfilled composite after bleaching. It was suggested that the increased reflectance was related to slight changes in translucency and particulate composition. It is noteworthy that reflections from a surface naturally decrease with increased roughness, but the lightness of a composite maybe a product of more complicated reflections related to translucency and subsurface particles such as fillers. Therefore the current results should be attributed to different structures and chemical compositions of the studied restorative materials, as discussed earlier.

Regarding b* values, bleaching resulted in a significant decrease in both materials subsequent to bleaching. It seems that the yellowness of the microfilled composite and giomer decreases subsequent to bleaching with 15% carbamide peroxide, which might be attributed to the effect of the agent on the pigments included in the composition, especially those on the surface. The results of the present study revealed that bleaching does not affect a* values in both materials remarkably and it can be concluded that within the limitations of this study bleaching with 15% carbamide peroxide did not affect redness and greenness of the restorative materials evaluated.

Under the limitations of the present study it can be concluded that the color stability of giomer, subsequent to bleaching, is comparable to that of microfilled composite resin. Therefore, giomer can be used as an appropriate restorative material in cervical areas in individuals with high caries activity, considering the ever-increasing use of bleaching agents, so that appropriate color stability can be achieved in addition to good esthetic results and anti-plaque properties. Although the color changes of microfilled composite resin and giomer were in the clinically acceptable range subsequent to bleaching, an increased surface roughness was observed in both materials.
